# QuPepFold: A python package for hybrid quantum-classical protein folding simulations with CVaR-optimized VQE

**DOI:** 10.1371/journal.pone.0342012

**Published:** 2026-02-11

**Authors:** Akshay Uttarkar, Vidya Niranjan, Amit Saxena, Vinay Kumar

**Affiliations:** 1 Department of Biotechnology, R V College of Engineering, (Affiliated to Visvesvaraya Technological University, Belagavi), Bangalore, Karnataka, India; 2 MIT Vishwaprayag University, Solapur, Maharashtra, India; 3 Centre for Development of Advanced Computing, Pune University Campus, Pune, Maharashtra, India; 4 School of Computer Science and Engineering, Presidency University, Itgalpur, Rajanakunte, Yelahanka, Bengaluru, Karnataka, India; Universite Cote d'Azur, FRANCE

## Abstract

**Background and Objective:**

Protein folding, and especially the conformational sampling of intrinsically disordered regions (IDRs), remains a formidable challenge for classical computation. We introduce QuPepFold, a modular Python package designed to democratize hybrid quantum–classical simulations of peptide folding, with the specific aim of enabling exploration of IDR ensembles for therapeutic targeting.

**Methods:**

We compute ground-state energies using a variational quantum eigensolver (VQE) that has been tuned with a conditional value-at-risk (CVaR) objective. This CVaR approach focuses on the lowest-energy measurement results, which speeds convergence and helps the algorithm cope with noise. The software provides an interface suitable for biologists and is independent of any particular quantum hardware; it currently runs on Qiskit Aer, Braket’s tensor-network simulator, and IonQ’s Aria-1 device through the Amazon Braket service.

**Results:**

In tests on short peptides up to ten amino acids long, the CVaR-optimized VQE reached the ground state roughly 30 percent faster than a standard VQE based on expectation values. When run on the IonQ Aria-1 quantum computer, it reproduced ground-state energies with over 90 percent fidelity. The agreement of results across simulators and physical devices indicates that the package yields consistent and transferable energies.

**Conclusions:**

QuPepFold offers an approachable yet extendable framework for integrating quantum techniques into peptide folding studies, particularly for sampling the ensembles of intrinsically disordered regions. By hiding the technical details of circuit construction and error mitigation, it lowers the barrier to using quantum computers in structural biology and opens opportunities for drug discovery against disordered proteins that have long been considered difficult to target.

## 1 Introduction

Proteins must fold into specific three-dimensional structures to perform their biological functions, and misfolding can lead to diseases ranging from neurodegeneration to cancer [1,2]. The classic “structure–function” paradigm holds that a protein’s amino acid sequence encodes a unique native structure, which in turn determines function [3]. However, a significant portion of the proteome defies this paradigm. Intrinsically disordered proteins (IDPs) and intrinsically disordered regions (IDRs) lack a stable folded structure under physiological conditions, instead existing as dynamic ensembles of conformations [3,4]. Recent estimates suggest that roughly one-third of eukaryotic proteins contain long disordered regions, and notably, about 79% of human cancer-associated proteins have at least one IDR [4]. These disordered proteins play crucial roles in cellular signaling, regulation, and phase-separated biomolecular condensates, and their dysregulation is linked to cancers, neurodegenerative disorders, diabetes and other diseases [5,6]. IDPs are thus increasingly recognized as important drug targets [6].

Despite their biomedical significance, IDRs were long deemed “undruggable” due to the absence of the well-defined binding pockets that structured proteins possess [4]. Traditional structure-based drug design relies on a stable 3D structure to identify binding sites for small molecules. By contrast, disordered proteins require alternative drug discovery strategies that account for their entire conformational ensemble rather than a single static structure [5]. Indeed, IDPs often undergo “coupled folding and binding” – folding into an ordered conformation only upon binding to a partner – resulting in transient, low-affinity interactions that challenge conventional lead design [5]. An accurate picture of the ensemble of conformations an IDR can adopt (and how this ensemble shifts upon binding) is essential for rationally targeting these proteins with therapeutics [5]. Experimental techniques like NMR, X-ray scattering, and single-molecule spectroscopy can probe aspects of IDP conformations, but they yield only averaged or partial information [6]. Computationally, simulating IDP ensembles is extremely demanding: the free-energy landscape of an IDP is relatively flat with many shallow minima separated by low barriers [3]. Exhaustively searching this vast conformational space requires traversing numerous degrees of freedom and highly accurate force fields [6]. Even with state-of-the-art molecular dynamics (MD) simulations on specialized hardware, capturing millisecond-timescale motions of IDPs incurs enormous computational cost [7]. In short, intrinsically disordered regions represent a major challenge for both experimental and in silico protein structure determination [3].

The limitations of current methods are exemplified by modern AI structure predictors: Deep learning approaches like AlphaFold2 have revolutionized protein structure prediction for ordered proteins [1], achieving atomic-level accuracy in many cases [2]. However, AlphaFold’s neural networks are trained on databases of folded structures and tend to output a single “most likely” conformation. Consequently, they struggle with IDPs that lack a unique structure [3]. AlphaFold can detect disorder (for instance via low confidence pLDDT scores in predicted models) and identify some segments that fold only upon partner binding [3] but it cannot fully capture the ensemble nature of IDR conformations [2,3] put it, AlphaFold “did not succeed” in reliably predicting intrinsically disordered protein structures. Thus, new computational paradigms are needed to address the protein folding problem in the context of IDRs. This is where quantum computing may offer a fresh advantage.

### 1.1 The protein folding problem and quantum computing potential

Protein folding has long been recognized as an extraordinarily complex optimization problem. Formally, even simplified versions (such as the hydrophobic-polar lattice model) are NP-hard, meaning the search for the global minimum energy conformation scales exponentially with protein length [8]. Classical approaches can manage small proteins or subdomains, but in general they cannot exhaustively explore the astronomically large conformational space of even mid-size proteins [9,10]. Techniques like physics-based molecular simulation face severe time-scale and sampling bottlenecks, while data-driven AI methods may falter when confronted with novel sequences, extensive mutation, or intrinsic disorder outside the training distribution [9]. Fundamentally, protein folding is governed by physics – the myriad atomic interactions that produce a complex, “rugged” energy landscape with many local minima [3]. Quantum computing is emerging as a promising tool to tackle such problems because it operates on different principles than classical machines. Quantum computers leverage superposition and entanglement to explore multiple states in parallel, and they naturally represent quantum mechanical systems, potentially allowing a more efficient sampling of energy landscapes (Perdomo-Ortiz et al., 2019). In principle, a suitable quantum algorithm could evaluate or traverse the protein conformational search space more effectively than a classical algorithm, by exploiting quantum tunneling or interference to avoid getting trapped in local minima. While practical quantum advantage in protein folding remains to be proven, the conceptual appeal is strong and has motivated intense research [10].

### 1.2 Quantum approaches to protein folding and IDRs

Recent quantum computing-based approaches to protein folding include quantum annealing, variational quantum eigensolvers (VQE), and the quantum approximate optimization algorithm (QAOA) [11]. Quantum annealing has been applied to small lattice models of protein folding on D-Wave systems with successful prediction of native conformations [11]. Gate-based quantum computing approaches like VQE have also been used to simulate low-energy conformations of short peptides [12]. Their comparative study of CVaR-VQE and MD simulations showed that quantum methods could find global minima more consistently than classical simulations [12]. [13] used IBM superconducting hardware to fold a 10-residue peptide using a resource-efficient VQE, marking one of the first biologically relevant folding predictions using quantum computers. Fingerhuth, Babej, and Ing (2018) [14] applied QAOA to lattice models, demonstrating how quantum algorithms can encode hard constraints for folding. Doga et al. (2023) [19] further argue that quantum computing is especially well-suited for folding disordered or mutated regions where AlphaFold performs poorly, providing early proof-of-concept on Zika virus IDR loops.

### 1.3 Toward a quantum advantage in drug discovery

Quantum computing may revolutionize drug discovery by enabling structure prediction for targets previously considered undruggable due to disorder. Romero et al. (2025) [10] recently folded 12-residue peptides on a 36-qubit trapped-ion quantum processor using a bias-field digital counterdiabatic QAOA, the largest protein-like system folded on real hardware to date. This breakthrough illustrates the rapid progression toward drug-relevant use cases and highlights the need for hybrid quantum-classical workflows. These can help refine flexible loops, evaluate ligand binding, or simulate ensembles for docking. The integration of quantum modules into Python-based tools for peptide-IDR interaction modeling represents a timely opportunity for the field.

### 1.4 A need for an automated tool and package

While recent breakthroughs in quantum computing for protein and peptide folding are promising, the field currently lacks standardized, accessible platforms for researchers to harness quantum resources in a practical, repeatable manner. Existing quantum algorithms, whether annealing-based or gate-based (e.g., VQE, QAOA), have been tested largely in isolated, experimental studies with limited portability, and often require deep domain knowledge of quantum programming frameworks like Qiskit, PennyLane, or Cirq [15]. As a result, there is a significant entry barrier for biophysicists, structural biologists, and drug discovery researchers who wish to explore quantum-enhanced folding simulations without a strong background in quantum computation [16].

Furthermore, current open-source quantum chemistry libraries focus primarily on small molecule simulation (e.g., Qiskit Nature, OpenFermion, and Psi4), offering little to no support for coarse-grained or residue-level protein folding—especially for disordered peptides or non-globular protein fragments that lack stable tertiary structure [17]. Tools like AlphaFold and Rosetta are state-of-the-art in classical peptide modeling but fall short when asked to simulate highly flexible, thermodynamically shallow ensembles typical of intrinsically disordered regions (IDRs) [18–20]. The integration of quantum solvers into peptide modeling pipelines is still in its infancy.

An automated Python package dedicated to quantum-enabled peptide folding would fill a critical gap in this space. Such a tool should serve as an abstraction layer that connects biological input (e.g., FASTA sequences of peptides) with quantum-native algorithms and simulators, enabling streamlined execution on both classical quantum simulators and real QPUs.

Such a toolkit would democratize quantum protein folding research by hiding the complexities of circuit design and quantum noise mitigation behind clean, biologically meaningful results.

In this study, we developed a reproducible, quantum-enhanced computational framework to simulate the folding behavior of short peptides (up to 10 residues), including disordered fragments, using Variational Quantum Algorithms (VQAs). These include the Variational Quantum Eigensolver (VQE) and a Conditional Value-at-Risk (CVaR)-optimized variant. Our method builds upon and extends the design space of lattice-based peptide folding, translating molecular constraints into quantum hamiltonians. We map the discrete tetrahedral-lattice conformational space onto a qubit register, where each computational basis state encodes a specific backbone-turn configuration and contact pattern. This approach was implemented as a modular Python package, QuPepFold, offering direct execution across simulators (Qiskit Aer, Braket TN1) and real quantum hardware (IonQ Aria-1).

The methodology integrates both theoretical modeling and implementation-level considerations, enabling systematic experimentation, benchmarking, and visual interpretation of quantum folding outcomes.

## 2 Methodology

### 2.1 Biophysical model and hamiltonian formulation

The native state of a protein corresponds to its thermodynamic ground state. We express this mathematically as a global energy minimum of a physically inspired Hamiltonian.


$$H(q)=H_{\text{gc}}\left(q_{\text{cf}}\right)+H_{\text{ch}}\left(q_{\text{cf}}\right)+H_{\text{in}}\left(q_{\text{cf}},q_{\text{in}}\right)$$ (1)

Here,

$H_{\text{gc}}$ enforces no “back-folding” (geometrical growth constraints),

$H_{\text{ch}}$ imposes correct L-chirality of side chains, and

$H_{\text{in}\;}$ encodes all bead-bead interactions through additional “contact” qubits $q_{\text{in}\;}$

For each non-bonded residue pair i,j and allowed lattice separation 𝓁, we introduce a binary contact variable $q_{ij\text{𝓁}}\in \{\text{0},\text{1}\}$ indicating whether residues i and j are in contact at separation 𝓁. For brevity we denote these contact variables as $q_{\text{cf}}$ in Eq. (1).

For each pair of beads (i,j) whose lattice separation is d(i,j)=𝓁, introduce a contact qubit $q_{ij}^{\left(\text{𝓁}\right)}$ and define


$$H_{in}^{\left(i,j,l\right)}\;=\;q_{ij}^{\left(l\right)}\left[\in_{ij}^{\left(l\right)}+\lambda \left(d\left(i,j\right)-l\right)\right]$$ (2)

When $q_{ij}^{\left(\text{𝓁}\right)}=\text{1}$ and d(i,j)=𝓁, the system gains the stabilizing energy $\in_{ij}^{\left(\text{𝓁}\right)}$; if the distance doesn’t match, the penalty λ≫∈ discourages assignments with $q_{ij\text{𝓁}}=\text{1}$ when the decoded lattice distance satisfies $d_{ij}(𝐛)\neq \text{𝓁}$.”.

**Fig 1:**
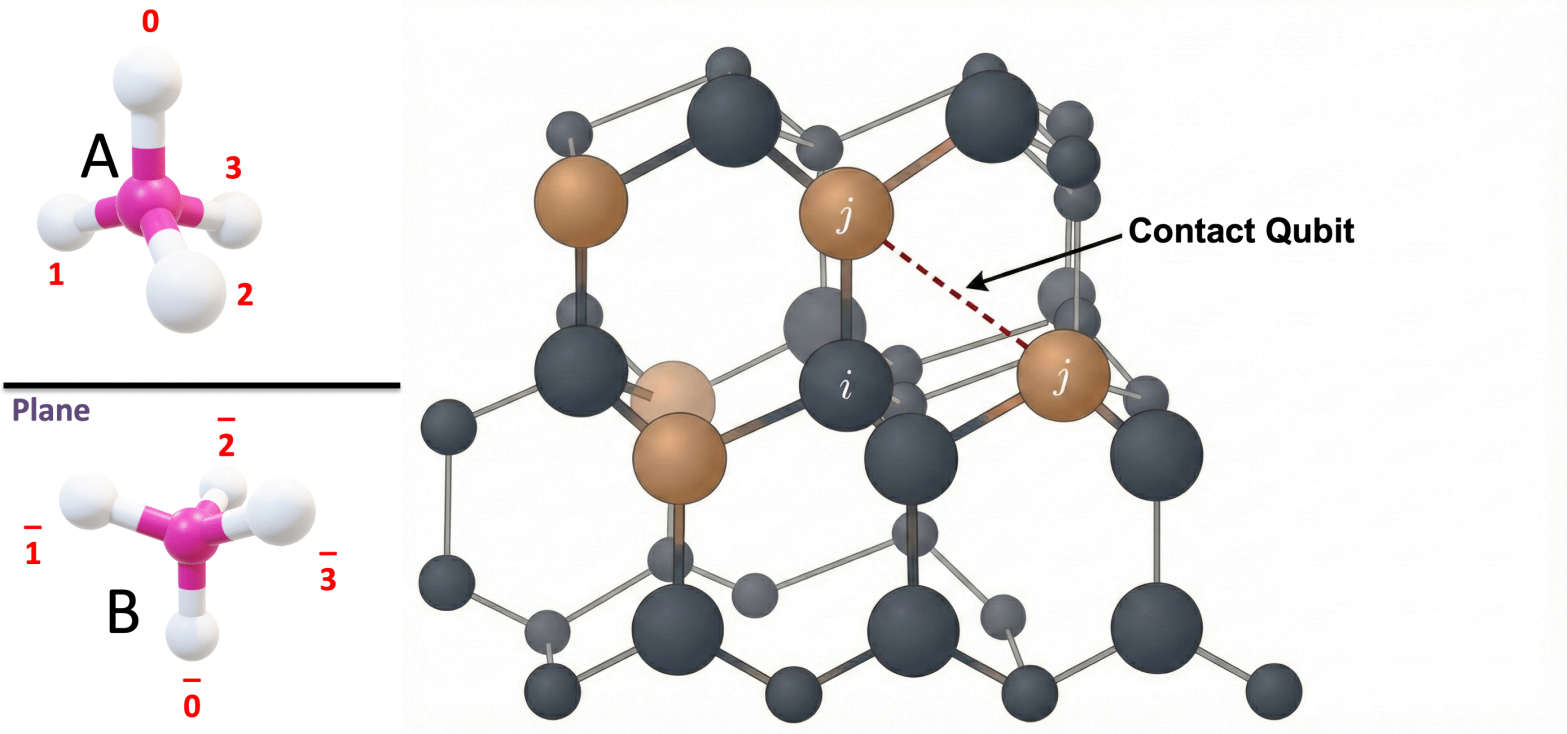
Schematic representation of a peptide on the tetrahedral lattice. Residues are shown as beads at lattice nodes. A non-bonded pair of beads (i,j) is highlighted, with its corresponding contact qubit q indicated, clarifying the assignment for each such pair.

Distance here refers to the bond length and more technically, for a given bitstring 𝐛, we decode the lattice configuration and compute the lattice distance $d_{ij}$ (b) between residues i and j. When $d_{ij}(𝐛)$ equals the encoded separation 𝓁 associated with a contact qubit $q_{ij\text{𝓁}}$, the system gains the stabilizing contact energy. When $d_{ij}(𝐛)\neq \text{𝓁}$, a penalty term is added, discouraging this inconsistent contact assignment. He tetrahedral assignment and distance measured as a schematic is shown in Fig 1.

The key innovation lies in the residue-level contact encoding, where nonbonded residue pairs *(i, j)* are coupled through interaction qubits, associated with distances, and scored using the Miyazawa–Jernigan (MJ) statistical potential.

Eq. (3) is obtained by summing the pairwise contact term of Eq. (2) over all non-bonded residue pairs i<j and all allowed lattice separations 𝓁, with $E_{ij\text{𝓁}}^{\text{MJ}}$ drawn from the Miyazawa-Jernigan matrix.


$$H_{i,j,\text{𝓁}}^{\left(\text{int}\right)}=q_{ij}^{\left(\text{𝓁}\right)}\cdot \in_{ij}^{\left(\text{𝓁}\right)}+\lambda \cdot (d(i,j)-\text{𝓁}{)}^{\text{2}}$$ (3)

Where:

$q_{ij}^{\left(\text{𝓁}\right)}\in \left\{\text{0},\text{1}\right\}$: Qubit indicating contact between residues i and j at distance 𝓁

$\in_{ij}^{\left(\text{𝓁}\right)}$: MJ-derived energy

λ: Penalty weight ensuring physical distance consistency

### 2.2 Lattice discretization and qubit encoding

We discretize the 3D conformational space using a tetrahedral lattice model, where each amino acid’s position is specified by a local turn encoding. The directional turns (e.g., right, left, up, down) are mapped into binary strings, and a full conformation is defined by a sequence of such binary codes. The turn encoding follows a one-hot or dense mapping based on sequence length

Sparse encoding requires


$$N_{\text{cf}}=\text{4}(N-\text{3}),$$ (4)

since each of the (N−1) turns (excluding fixed endpoints) is encoded by four one-hot qubits, minus eight qubits for the two fixed initial turns.

Dense encoding reduces this to


$$N_{\text{cf}}^{\text{dense}\;}=\text{2}(N-\text{3})$$ (5)

using two qubits per turn via a binary scheme, halving the qubit overhead at the cost of higher-locality interactions.

Equations 4 and 5 quantify how many qubits are needed just to record the backbone (and sidechain) turns of an N-residue peptide on the lattice.

For example, a 7-residue peptide requires at least 6 turns (or 12 qubits in dense encoding) and an additional 3–5 interaction qubits, yielding circuits with ~15–18 total qubits. The encoding qubits specify backbone geometry, while interaction qubits control tertiary contacts.

In QuPepFold, this is implemented through the generate_turn2qubit() function, which returns valid conformational states under symmetry reduction (e.g., fixing initial bits like 00, 01, 10) and lattice connectivity constraints.

### 2.3 Quantum Hamiltonian construction

Each constraint term (geometry, chirality, contacts) is translated into Pauli operators using Qiskit’s PauliSumOp. The MJ potential is handled via a lookup matrix, implemented in build_mj_interactions(), that assigns energies to residue-residue pairs and builds corresponding Pauli expressions.

Additional constraints—such as non-overlap and chain connectivity—are encoded as ancilla-based or penalty terms with quadratic scaling. The full Hamiltonian is assembled dynamically for any sequence.

### 2.4 Variational ansatz and quantum circuit compilation

**Fig 2:**
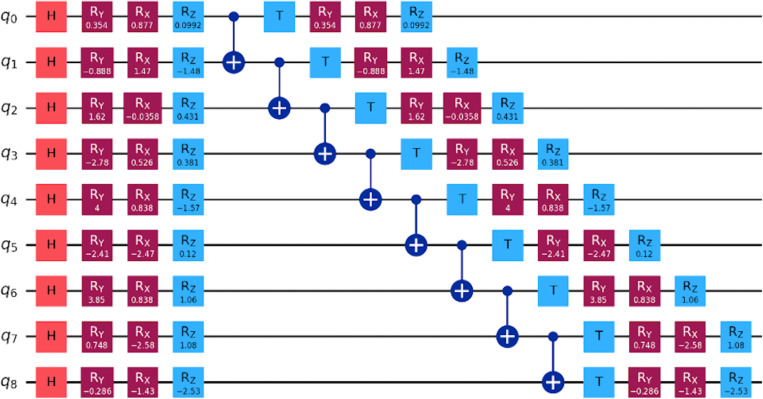
A circuit design for a 7 amino acid peptide sequence.

To solve the Hamiltonian minimization, we employ a hardware-efficient VQE ansatz, initialized with first, a single-qubit parameterized gates: $R_{x}(\theta ),R_{y}(\varphi ),R_{z}(\gamma$ followed by entangling CNOT blocks which is implemented in linear or circular topology. In the next step we use, T gate to enforce constraint-aware exploration. A circuit design for a 7 amino acid peptide sequence is provided in Fig 2.

These gates form layered quantum circuits with variable depth, controlled by user input. For short peptides (≤10 residues), a 3-layer ansatz sufficed. The circuit is built in the protein_config_ansatz() class and compiled using Qiskit’s transpiler for depth minimization.

To ensure NISQ compatibility, compiled circuits are verified against maximum hardware thresholds (20 qubits, 60–80 depth). The pipeline supports backend-agnostic execution via Qiskit and Amazon Braket interfaces. In our previous experimental results [21,22] on the IonQ Aria-1 hardware, where CVaR-VQE circuits of comparable scale achieved >90% fidelity in ground-state energy estimation for short peptides.

### 2.5 Objective function and optimization: CVaR-VQE

Using the parameterized ansatz circuit generated by protein_config_ansatz(), we translate the combinatorial optimization over bitstrings into an optimization over the rotation angles θ. Gate-based quantum devices do not allow direct search over discrete bitstrings. Instead, a parameterized ansatz prepares a probability distribution over bitstrings, controlled by continuous rotation angles θ. By optimizing these angles to minimize the measured energy, we effectively translate the discrete optimization problem into a continuous parameter optimization compatible with current quantum hardware.

These angles have nothing to do with the angles formed by the protein beads. The target function’s set of angles is used by the quantum circuit to create a range of bit strings with varying probabilities. The target function is defined as weighted average of the smaller energies and invoke the exact Hamiltonian function on the bit strings that the quantum circuit provided. For the disordered region peptide, there were 100 iterations total.

More specifically, the corresponding probabilities are ranked by energy after the energy for each observed fold is computed. An anticipated energy that is calculated from the tail end of the probability distribution and cutoff by an alpha parameter is returned by the objective function. A conditional value at risk is this expectation energy (CVaR).


$${𝐂𝐕𝐚𝐑}_{\alpha }\left(\theta )=\langle \psi (\theta )|𝐇(𝐪)|\psi (\theta \right)\rangle_{\alpha }$$ (6)

The average energy over the lowest α\alphaα-fraction of measurement outcomes. By focusing on the “tail” of low-energy samples, CVaR-VQE accelerates convergence compared to standard expectation-value minimization.

Tail-averaging biases the optimizer toward promising (low-energy) folds, reducing the number of circuit evaluations needed


$${\text{CVaR}}_{\alpha }(\theta )=\frac{\text{1}}{\alpha }\sum {\mathrm{\backslash nolimits}}_{i\in S_{\alpha }}\;p_{i}E_{i}$$ (7)

Where:

$S_{\alpha }$: Subset of outcomes with cumulative probability α (e.g., 5%)

$p_{i}$: Measured probability of bitstringi

$E_{i}$: Energy corresponding to bitstringi

Implemented in ProteinVQEObjective(), this metric ensures that optimization focuses on promising low-energy states, reducing sensitivity to noise and local minima.

### 2.6 QuPepFold architecture

The QuPepFold package is a modular, quantum-classical hybrid software toolkit built for peptide and protein folding simulations using quantum algorithms. Its primary goal is to provide a biologist-friendly yet quantum-complete platform that abstracts the complexity of quantum circuit design, Hamiltonian construction, and backend execution.

It supports both gate-based quantum computing (e.g., IBM Q, IonQ via Amazon Braket) and quantum simulators (e.g., Qiskit Aer, Braket TN1), making it extensible across current and near-future hardware.

The architecture follows a pipeline-based design, composed of independently callable modules with tight integration through a main interface fold(). The core workflow includes encoding, Hamiltonian assembly, ansatz building, objective evaluation (CVaR), and quantum execution.

QuPepFold consists of several tightly coupled modules that serve discrete responsibilities but communicate through a central execution kernel. The user initiates the folding pipeline by passing a peptide sequence along with optional configuration parameters such as the quantum backend, lattice encoding mode (dense or one-hot), penalty constants (λ), or a custom interaction potential. The input is processed by the generate_turn2qubit() module, which maps the 3D conformational space into a constrained binary configuration suitable for quantum encoding. This module supports lattice symmetry reductions and validates turn sequences against steric overlap rules to ensure physical plausibility. Following the geometric encoding, the build_mj_interactions()module loads or constructs a residue-residue interaction matrix, based on the statistical Miyazawa–Jernigan potential. It computes all plausible nonbonded contacts between residues and represents them as terms in a Hamiltonian expressed in qubit space.

The Hamiltonian is then assembled using the exact_hamiltonian() module, which supports both full symbolic construction and empirical pre-filtering of energetically insignificant terms. Each term is encoded using Pauli operators via Qiskit’s PauliSumOp, allowing seamless integration with quantum variational solvers. The protein_config_ansatz() module generates a parameterized quantum circuit using a hardware-efficient ansatz composed of rotation gates (Rx, Ry, Rz) and entangling layers (CNOT chains). This circuit is optimized iteratively using a optimizer in conjunction with the ProteinVQEObjective() module, which computes the Conditional Value-at-Risk (CVaR) of the measurement outcomes. CVaR ensures that optimization focuses on the lowest-energy conformational states, thus improving resilience to quantum noise and barren plateaus.

**Fig 3:**
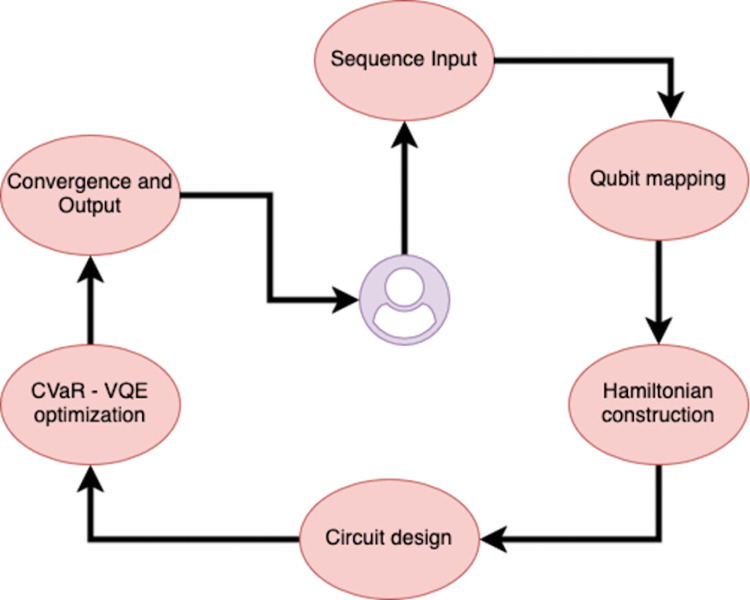
The schematic representation of the automated QuPepFold workflow.

The final execution is handled by the backend_manager() function, which allows users to deploy the same pipeline across Qiskit simulators (e.g., Aer), tensor-network simulators (e.g., Braket TN1), or real quantum hardware such as IonQ Aria-1. The results—bitstring distributions, convergence plots, and lowest-energy conformers—are returned as structured outputs (JSON/YAML), and visualizations are generated via the visualize_results() module. By encapsulating complex quantum logic behind user-friendly interfaces, QuPepFold provides access to quantum folding simulations, facilitating interdisciplinary adoption in computational biology and quantum drug discovery. The design is future-proof, allowing extensions such as side-chain modeling, hybrid classical-quantum loops, and ML-enhanced priors for conformational seeding. A schematic representation of the complete pipeline is provided in Fig 3.

The comprehensive list of the modular functions are provided in the Table 1

**Table 1:** The table with all module name in the package with respective functions.

Sl No	Module Name	Functionality
1	generate_turn2qubit()	Encodes 3D tetrahedral lattice turns to qubit strings (dense or one-hot encoding)
2	build_mj_interactions()	Loads the Miyazawa–Jernigan potential and defines pairwise residue energy terms.
3	exact_hamiltonian()	Computes classical Hamiltonian energy for benchmarking or hybrid validation
4	protein_config_ansatz()	Builds hardware-efficient variational ansatz circuits with rotation and CNOT layers
5	ProteinVQEObjective()	Defines the CVaR-based energy expectation evaluation for VQE optimization
6	fold(sequence, backend)	High-level API integrating all modules for full quantum folding simulation
7	visualize_results()	Plots convergence curves, CVaR histograms, and final folding bitstrings
8	backend_manager()	Selects and configures simulator or QPU (Qiskit, Braket TN1, IonQ)
9	config.yaml	Holds backend settings, sequence-specific options, and CVaR cutoffs

### 2.7 Mapping quantum bitstrings to 3D peptide PDBs

We convert high-probability quantum measurement outcomes (“bitstrings”) into deterministic, viewer ready 3D peptide structures. After executing the variational circuit and collecting measurement counts, bitstrings are ranked by empirical probability p(b)=counts(b)/∑_b_. Only outcomes with p(b)≥2% are exported. For each retained bitstring, we decode the backbone turn configuration from a template mapping (turn2qubit), infer a coarse secondary-structure (SS) trace from the local pattern of turns, and then build a full 3D backbone (N,CA,C,O) using fixed internal coordinates and SS-dependent (ϕ,ψ) targets. Minimal sidechains are added at Cβ for non-glycine residues. Each structure is written a PDB with HELIX/ SHEET annotations and explicit CONECT records (intra-residue bonds and peptide links) to ensure polymerization in all viewers [23]. Outputs are one PDB per bitstring plus a zipped bundle.

The circuit encodes a peptide of length N via a configuration segment of length 2(N-1), represented by a template string turn2qubit containing fixed bits (0/1) and placeholders (q). Given a measured bitstring b, we fill the q positions left-to-right to obtain a configuration bitstring b_cfg_. We parse b_cfg_ into consecutive 2-bit tokens t_j_∈{0,1,2,3} for j = 1,…,N-1, which encode relative turn classes produced by the ansatz. Any residual “interaction” bits beyond the configuration segment are ignored for geometry (they inform energy/contact scoring, not coordinate generation).

We transform the discrete turn sequence T=(t_1,…,t_(N-1)_) into a per-residue SS label ss_i_∈{H,E,C} using a two-stage heuristic designed for speed and determinism. First, a sliding window around residue *i* classifies regularity: uniform windows map to helix (H), and windows with consistent parity alternation (even/odd tokens alternating) map to strand (E); all other local patterns default to coil (C). Second, short H/E runs are suppressed (H < 4 residues, E < 3 residues →C) to reduce false positives. This yields a clean SS trace that controls backbone torsions without iterative refinement.

We build Cartesian coordinates by forward kinematics with ideal peptide geometry. Fixed bond lengths and angles are used for all residues (Å: C-N 1.329, N-CA 1.458, CA-C 1.525, C = O 1.229; angles in degrees: C-N-CA 121.7, N-CA-C 110.4, CA-C-N 116.2, CA-C-O 120.8). Peptide bonds are set trans ($\omega ={\text{180}}^{\circ }$). Dihedrals (φ,ψ) are assigned per residue from the SS label: helix ($-{\text{60}}^{\circ },-{\text{45}}^{\circ }$), strand ($-{\text{135}}^{\circ },{\text{135}}^{\circ }$), and coil ($-{\text{70}}^{\circ },{\text{140}}^{\circ }$). To avoid numerical degeneracy at the start, we seed ${\text{N}}_{\text{1}}$ at the origin, ${\text{CA}}_{\text{1}}$ on +x at 1.458Å, place ${\text{C}}_{\text{1}}$ in the xy-plane to satisfy ∠N−CA−C, and position ${\text{O}}_{\text{1}}$ using the CA-C-O angle. Each subsequent atom is placed via a robust local frame derived from the previous three atoms (A,B,C): we construct a right-handed $\left(\overset{\circ }{x},\overset{\circ }{y},\overset{\circ }{z}\right)$ at C and compute $\vec{CD}$ from (𝓁,θ,χ) using standard internal-to-Cartesian formulas. If cross-products approach zero (nearly colinear vectors), an orthogonal fallback direction is injected to maintain frame stability and prevent NaNs.

For non-glycine residues, we place a single sidechain atom Cβ to convey sidechain orientation and aid visual interpretation. From the local backbone frame at CA, we form ${\overset{\circ }{u}}_{\text{1}}=\overset{―}{\text{CA}\to \text{N}}$ and ${\overset{\circ }{u}}_{\text{2}}=\overset{―}{\text{CA}\to \text{C}}$, compute $\overset{\circ }{u}=\text{normalize}\left({\overset{\circ }{u}}_{\text{1}}+{\overset{\circ }{u}}_{\text{2}}\right)$ and peptide-plane normal $\overset{\circ }{n}=\text{normalize}\left({\overset{\circ }{u}}_{\text{1}}\times {\overset{\circ }{u}}_{\text{2}}\right)$, then place CB=CA+1.53 normalize $\left(\text{0}.\text{943}\overset{\circ }{u}+\text{0}.\text{333}\overset{\circ }{n}\right)$Å. This tetrahedral-like construction yields a sensible off-plane Cβ without invoking full rotamer libraries.

For each retained bitstring, we write ATOM records for N,CA,C,O (and CB if non-Gly) with standard three-letter residue names on chain A, residues 1..N. The SS trace is encoded as HELIX records for H runs ≥4 and SHEET records for E runs ≥3. To ensure correct polymerization across viewers that do not infer bonds, we emit explicit CONECT records: intra-residue links N−CA,CA−C,C−0, plus CA−CB when present; and inter-residue peptide links C(i)−N(i+1). A remark bitstring c_fg_ bits embeds the configuration used. Structures are written to pdb3d/ and bundled in a single zip archive.

All constants and thresholds are fixed for determinism and reproducibility parameters are mentioned in the Table 2.

**Table 2:** Comprehensive reproducibility parameters for peptide folding.

Parameter	Value (default)	Rationale
Export threshold (τ)	0.02	Match histogram “≥ 2%”
Bond lengths (C–N, N–CA, CA–C, C = O)	1.329, 1.458, 1.525, 1.229 Å	Ideal peptide geometry
Angles C–N–CA, N–CA–C, CA–C–N, CA–C–O	121.7°, 110.4°, 116.2°, 120.8°	Ideal peptide geometry
ω (peptide dihedral)	180°	Trans peptide bonds
(φ, ψ)_H	(−60°, −45°)	α-helical mean
(φ, ψ)_E	(−135°, + 135°)	β-strand mean
(φ, ψ)_C	(−70°, + 140°)	Extended coil
Cβ distance	1.53 Å	Typical CA–CB
H/E minimum run length	4/ 3	Stability of SS records
Degeneracy tolerance (ε)	1 × 10 ⁻ ⁸	Frame stability

## 3 Results

The execution pipeline of the QuPepFold Python package operationalizes a variational quantum-classical hybrid framework tailored for protein and peptide folding, specifically addressing short peptide sequences and intrinsically disordered regions (IDRs). The design is modular and scalable, enabling deployment across a variety of quantum backends, including Qiskit simulators, Amazon Braket’s tensor network engine (TN1), and real quantum processing units (QPUs) such as IonQ Aria-1. This section details the functional structure and scientific logic embedded in the core execution modules, highlighting the mathematical, algorithmic, and biophysical principles involved.

### 3.1 Conformational encoding into qubit register

The execution begins with geometric abstraction of the input peptide sequence using a tetrahedral lattice model. The generate_turn2qubit() module converts an amino acid sequence of length NN into a binary representation of directional turns. The number of required turns is calculated as 2(N − 1) accounting for three-dimensional spatial transitions between adjacent residues. A subset of qubits termed “fixed bits” is preassigned to canonical initial directions to eliminate symmetry-related redundancies. The remaining qubits “variable bits” encode permissible conformational changes and are subject to quantum optimization.

This step is essential for reducing the peptide folding problem into a finite, discrete conformational space that can be evaluated via quantum measurements. The register effectively represents a coarse-grained folding space where each bitstring corresponds to a unique spatial arrangement of the backbone.

### 3.2 Interaction potential initialization via MJ matrix

Residue-residue interactions are parameterized using a modified Miyazawa–Jernigan (MJ) contact potential matrix [24], initialized within the build_mj_interactions() module. This matrix captures statistical contact preferences between all 20 canonical amino acids, ensuring that hydrophobic, electrostatic, and aromatic tendencies are embedded in the energetic landscape.

The MJ matrix is symmetrized and scaled with a negative bias to reflect favorable interactions energetically. For each pair of residues *i* and *j,* the effective interaction energy *(i,j)* is retrieved and mapped into the Hamiltonian. While the current implementation generates randomized MJ values for demonstration, future versions of the package will support user-defined or empirical potentials, enabling integration of experimental data or force-field corrections.

### 3.3 Hamiltonian formulation and energy evaluation

The exact_hamiltonian() function computes the total folding energy associated with a given set of qubit-derived bitstrings. The Hamiltonian is defined as a weighted sum of several biophysically grounded penalty terms:


$$H(q)=\lambda_{\text{dis}\;}\cdot E_{\text{dist}\;}+\lambda_{\text{loc}\;}\cdot E_{\text{local}\;}+\lambda_{\text{back}\;}\cdot E_{\text{backfold}\;}+H_{\text{MJ}\;}$$ (8)

Where:

$E_{\text{dist}\;}$: penalizes geometric violations in residue distances,

$E_{\text{local}\;}$: penalizes invalid local conformations,

$E_{\text{backfold}\;}$: penalizes steric clashes and loop overs,

$H_{\text{MJ}}$: aggregates pairwise MJ interaction energies.

Each candidate bitstring is decoded into a 3D structure, and inter-residue distances are computed. These are compared against idealized lattice distances, with deviations contributing to the energy via quadratic penalties. This classically evaluated energy acts as both a validation benchmark and a target for variational optimization

### 3.4 Variational ansatz construction

The conformational search is operationalized through a quantum variational algorithm. Although not shown explicitly in the previewed code, the package internally constructs a hardware-efficient ansatz using the Qiskit QuantumCircuit object. Each ansatz layer includes parameterized rotation gates *R*_*x*_(θ),*R*_*y*_(ϕ),*R*_*z*_*(γ*) followed by entangling gates (e.g., CNOTs) configured in linear or circular topology.

The number of layers and parameter sets is user-tunable, and transpilation is applied to conform to backend-specific constraints such as maximum depth or gate fidelity thresholds. The ansatz explores the high-dimensional Hilbert space of folding configurations, searching for variational parameters that minimize the expectation value of the Hamiltonian

### 3.5 Objective function: *Conditional value-at-risk* (*CVaR*)

The optimization is guided not by the average energy, but by the Conditional Value-at-Risk (CVaR), a statistical risk metric that focuses only on the lower tail of the energy distribution. Formally:


$${\text{CVaR}}_{\alpha }(\theta )=E\left[E_{i}\;|\;E_{i}\leq {\text{VaR}}_{\alpha }\right]$$ (9)

Here, α\alphaα is the risk threshold (e.g., 0.05), and the expectation is taken over only the lowest-energy outcomes E_*i*_. This enhances convergence by ignoring high-energy, noise-prone bitstrings and targeting the subset most likely to represent viable conformations. This approach is especially effective in near-term quantum devices with limited coherence and high gate error rates.

### 3.6 Output generation and visualization

**Fig 4:**
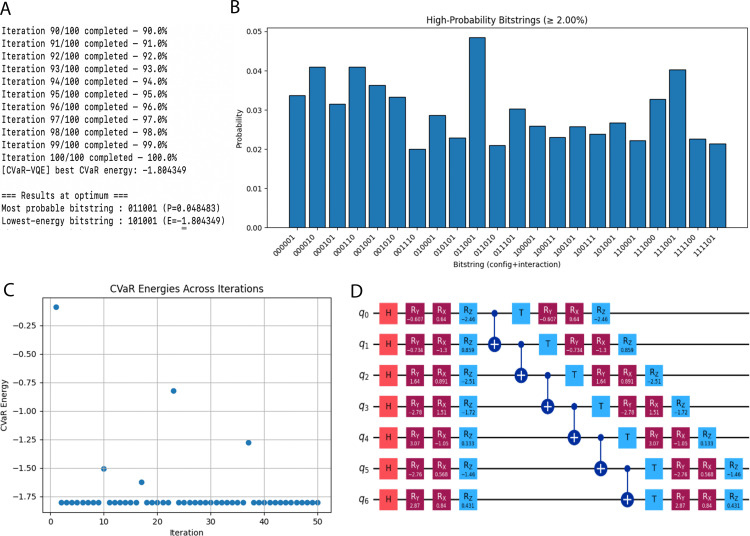
Example output plot. **A)** The partial terminal output after running for 100 iterations with expected output. **B)** A bar chart with bitstrings (folds) with probability in y-axis. **C)** Convergence plots of CVaR energy over iterations. **D)** The ansatz circuit generated for the calculation.

The output consists of the following files. 1. Terminal out with C-VaR energy (Fig 4A), most probable bitstring, lowest energy bitstring. 2. Histograms of measurement frequencies (Fig 4B). 3. Convergence plots of CVaR energy over iterations (Fig 4C), 4. The ansatz circuit generated for the calculation (Fig 4D).

**Fig 5:**
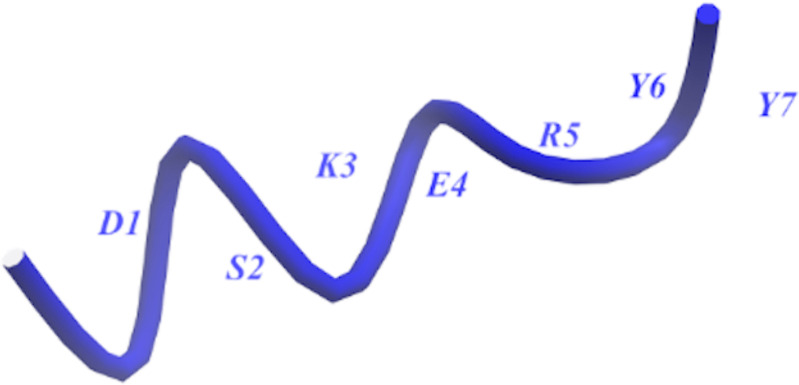
The ribbon representation of the peptide used for the bitstring 101000 from Fig 4A (most probable) folding pattern. The amino acid sequence along with residue number in shown.

An example visualization for the sequence DSKERYY is provided of the top-ranked folding bitstrings and the ansatz is provided in Fig 4 and the peptide folding visualization for the most probable bitstring is provided in Fig 5.

These are visualized using matplotlib in a self-contained plotting module. Additionally, the output layer supports parsing of bitstrings back into structural conformations for downstream 3D modeling or comparison with classical molecular dynamics (MD) data.

### 3.7 Prerequisites and working environment

The package is designed to provide a modular and backend-agnostic platform for simulating peptide folding via variational quantum eigensolvers. Owing to the computational complexity of Hamiltonian construction, qubit encoding, and quantum circuit optimization—especially when applied to biologically relevant sequences—the package requires a robust Python environment and sufficient system resources to execute simulations efficiently.

Table 3 outlines the core software dependencies and supported quantum development kits (SDKs) required to run the package. The implementation relies heavily on numpy and scipy for numerical operations, matplotlib and seaborn for visualization, and pyyaml for configuration parsing. Backend compatibility is ensured through optional integration with Qiskit (for local or IBM quantum backends) and Amazon Braket (for access to the TN1 tensor-network simulator and the IonQ Aria-1 QPU).

**Table 3:** Comprehensive table on the working prerequisites for QuPepFold.

Category	Requirement	Details/Commands
Python Version	Python ≥ 3.8	Recommended to use with venv or conda environments Simple usage: run pip3 install QuPepFold
Core Python Packages	numpy, scipy, matplotlib, pyyaml, tqdm	Install via: pip install numpy scipy matplotlib pyyaml tqdm
Quantum SDK – Qiskit	qiskit	For Aer simulator and IBMQ: pip install qiskit
Quantum SDK – Braket	amazon-braket-sdk, amazon-braket-default-simulator	For TN1 simulator and IonQ QPU: pip install amazon-braket-sdk amazon-braket-default-simulator
Optional Tools	seaborn, jupyterlab	For enhanced plots and notebook use
Hardware (Recommended)	Internet connection, AWS credentials	Required for Braket backend (~/.aws/credentials)

Additionally, users are encouraged to install JupyterLab or equivalent notebook environments for interactive usage and iterative debugging, particularly when tuning variational parameters or visualizing convergence plots.

Table 4 details the minimum and recommended hardware specifications required to run QuPepFold. The resource footprint scales with peptide length, number of qubits, and backend complexity. For instance, a 10-residue peptide typically requires 20–25 qubits, leading to dense Hamiltonians composed of several thousand Pauli terms. As such, systems with at least 16 GB of RAM are required for stable execution, while 32 GB or higher is recommended for simulations involving larger peptides or ensemble runs.

**Table 4:** The table with minimum requirements to run QuPepFold.

Component	Minimum Requirement	Recommended for Large Peptides.
CPU	Quad-core 64-bit	8-core CPU (Intel i7/Ryzen 7 or equivalent)
RAM	≥16 GB	≥32 GB for large Hamiltonians and multi-bitstring simulations
Storage	≥2 GB free space	SSD recommended for faster cache and data writes
GPU (Optional)	Not required	CUDA-compatible GPU for acceleration (visualization only)
Internet	Required for remote execution	Stable high-speed internet for AWS Braket/IonQ integration
Cloud Access	AWS Account with Braket enabled	Needed for IonQ and TN1 execution

Moreover, while local simulations (using Qiskit Aer) are feasible on high-end laptops, cloud-based simulations—especially via Braket’s TN1 simulator or real QPUs—require an active internet connection, authenticated access to AWS services, and potentially enhanced compute capacity to manage upload and retrieval of circuit batches.

### 3.8 Testing on simulator (Aer) and benchmarking

We benchmarked QuPepFold on a diverse panel of short peptides derived from intrinsically disordered protein–like sequences, spanning lengths from N = 6 to N = 10. The dataset comprises 1,224 sequences generating 21,600 conformers.

For N = 6, the query contained 244 sequences. The algorithm proved highly effective. 99.6% of sequences successfully reached a negative energy state, indicating that the fixed budget is sufficient to find stabilized folds in this regime. The mean best energy was −2.97 kcal/mol, and the method-maintained diversity by exploring ~1.98 unique geometries per sequence.

For N = 7, the query included 258 sequences and it yielded the strongest stability results. The mean best energy deepened to −5.57 kcal/mol, with 97.3% of sequences finding negative energy minima. This length represents a best optimal peptide length where the algorithm consistently locates the lowest-energy geometric basins (1.06 geometries/sequence) efficiently (7.45 s/sequence).

For N = 8 we had 172 sequences in the query, the search space expands significantly. While the mean best energy shifted to positive values (57.21 kcal/mol) due to the limited sampling budget, the focus remains on the 13.4% of sequences that successfully reached negative energy states. This subset demonstrates that QuPepFold can still locate stabilized folds in larger spaces, though the bulk of positive energy outcomes. The search collapsed to a single dominant geometry per sequence, with structure explaining ~74% of the energy variance.

For N = 9, there were 150 sequences, and the mean energy was at 63.80 kcal/mol. Although most low-budget runs yield positive energies, the algorithm is expected to identify negative-energy stabilized folds in approximately 4.7% of cases. The geometry-energy correlation tightens (R2 ≈ 0.81), suggesting that finding the correct geometry is the primary bottleneck to achieving negative energy.

For N = 10 with 135 sequences the trend continues with a mean energy of 70.45 kcal/mol. The success rate for locating negative energy states is estimated at 1.5% under the current budget. Despite the prevalence of positive values, these rare negative-energy instances are the critical result, validating that the quantum-hybrid pipeline can theoretically access the global minimum even when the landscape is dominated by high-energy decoys. The results are consolidated in Table 5.

**Table 5:** A comparative table showing the benchmarking results of Qupepfold.

Length (N)	N_Seq	Conformers generated	Unique Geometries per Seq	R² (Geometry vs Energy)	% Sequences with Negative Energy	Runtime/iteration (s)
6	244	483	1.98	0.160	99.6%	4.66
7	258	273	1.06	0.313	97.3%	7.45
8	172	172	1.00	0.739	13.4%	10.48
9	150	150	1.00	0.812	4.7%	13.31
10	135	135	1.00	0.884	1.5%	16.22

**Fig 6:**
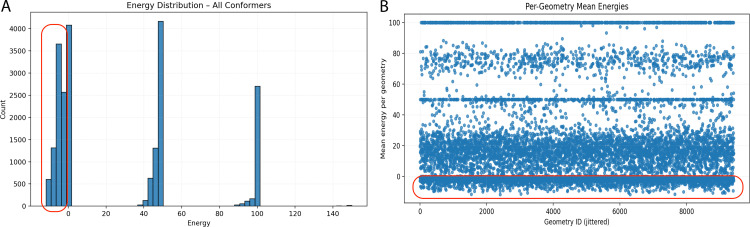
Analysis of energy landscapes across generated conformers. **(A)** Histogram illustrating the frequency distribution of energy values for all conformers, highlighting a distinct multimodal distribution with primary clusters centered near 0, 50, and 100 energy units (kcal/mol). **(B)** Scatter plot depicting the mean energy per geometry (with jitter applied to Geometry ID for visibility), revealing horizontal stratification that corresponds to the discrete energy populations observed in the histogram.

To better understand the stability and energetic feasibility of the generated structures, we analyzed the energy profiles of the entire conformer library. As shown in Fig 6A, the energy distribution is not continuous but rather multimodal, segregating into three distinct populations.

The majority of the conformers occupy a low-energy basin centered slightly below (<0 kcal/mol, within the red zone), representing the most energetically favorable and stable geometries. A second, clearly defined population appears in the intermediate range of approximately 40–50 energy units. Interestingly, there is a sharp, high-density peak at the 100-mark. In the context of this simulation, such a distinct high-energy cutoff suggests a subset of conformers that either encountered steric clashes or were assigned a penalty value during the optimization process, effectively grouping them into a “high-energy” cluster.

This discretization of energy states is further elucidated in Fig 6B, which maps the mean energy against specific geometry IDs. Rather than a random scatter, the data exhibits clear horizontal banding. The dense concentration of points near the baseline (y < 0) confirms that a significant portion of the distinct geometries consistently achieve stable energy scores, marked within the red zone. Conversely, the bands at y ≈ 50 and y ≈ 100 indicate that specific geometric scaffolds inherently predispose the conformers to higher energy states, regardless of minor structural fluctuations. This stratification implies that the structural diversity of the dataset is coupled with discrete energy levels, effectively filtering the geometries into stable, metastable, and unstable categories.

## 4 Discussion

QuPepFold employs variational quantum eigensolver (VQE) algorithms with a conditional value at risk (CVaR) aggregator to explore peptide conformations. It encodes the conformational space of short peptides (2–10 amino acids) into qubits and optimizes a parameterized quantum circuit (ansatz) to find low-energy structures. This hybrid quantum–classical approach iteratively measures energy and updates circuit parameters, focusing on the lowest-energy outcomes via CVaR to bias the search towards folded conformations. In doing so, QuPepFold emphasizes ground-state energy search (global minima on the energy landscape) using shallow circuits compatible with noisy intermediate-scale quantum (NISQ) devices.

The optimization of variational parameters in QuPepFold does not rely on an external training dataset of protein structures. Instead, it follows the standard variational quantum eigensolver (VQE) paradigm: the parameters are learned on-the-fly by minimizing the expectation value of the Hamiltonian for the peptide under study.

The parameterized circuit is defined by a vector of angles $\theta \in [-\pi ,\pi {]}^{d}$, where each component controls a rotation gate acting on either configuration or interaction (ancilla) qubits. These angles are unrelated to physical dihedral angles of the peptide; they are purely variational knobs in the quantum circuit. For a given θ, the circuit is executed repeatedly to generate a distribution of bitstrings b with empirical probabilities p(b). For each observed bitstring, the exact classical Hamiltonian is evaluated via exact_hamiltonian() to obtain an energy E(b).

Rather than minimizing the simple expectation value $\sum_{b}\;p(b)E(b)$, QuPepFold employs a Conditional Value-at-Risk (CVaR) objective implemented in the ProteinVQEObjective() module. The bitstrings are sorted by energy, the lowest-energy tail of the distribution is selected up to a user-defined cumulative probability α, and the CVaR is defined as the average energy over this tail. By focusing on the lowestenergy fraction of outcomes, the optimizer is biased toward promising folds and becomes less sensitive to noise in higher-energy samples. In practice, ProteinVQEObjective() uses a reduced cutoff (e.g., α=0.025 in noiseless simulations and α=0.05 on hardware).

In contrast, QFold [25] combines quantum walks with a Metropolis algorithm guided by deep learning. Rather than relying on lattice models, QFold parameterizes conformations by continuous torsion angles, using quantum walks to sample moves in torsion space. This off-lattice strategy is more realistic for protein and peptide backbones, avoiding the geometric simplifications that other methods often require. QFold’s hybrid algorithm does not depend on VQE or the quantum approximate optimization algorithm (QAOA); instead, it implements a quantum-enhanced Metropolis sampling. A minimal version of QFold’s quantum Metropolis algorithm has been run on IBM quantum hardware (Casablanca) to validate the concept, demonstrating feasibility for very small peptides [25]. Other efforts have applied QAOA to peptide folding as a combinatorial optimization problem. Boulebnane et al. (2023) [26] encoded each backbone turn on a tetrahedral lattice into qubits and applied QAOA to identify low-energy conformations. Although QAOA offers a structured p-layer alternation of problem and mixer Hamiltonians, near-term hardware limitations mean accurate folding requires deep circuits. At low circuit depths, its performance did not exceed random sampling, underscoring the challenge of using QAOA for realistic folding tasks on NISQ devices.

Quantum annealing provides an alternative paradigm. Perdomo-Ortiz et al. (2012) [27] mapped small lattice protein models (hydrophobic–polar HP models) to a D-Wave quantum annealer, solving folding problems with up to 81 qubits. This approach treats folding as a quadratic unconstrained binary optimization problem and exploits quantum tunneling to escape local traps. Despite its pioneering nature, the method relies on drastic problem simplifications and mappings that scale exponentially with chain length. More recent studies, such as Irbäck et al. (2024) [28], have used D-Wave’s hybrid solvers to handle HP model chains up to 64 residues, but pure quantum annealing still struggles as problem size increases.

A recent innovation is the digitized-counterdiabatic (dCD) algorithm developed by Chandarana et al. (2022) [29]. This method extends VQE by incorporating counterdiabatic terms to shorten the adiabatic path. In experiments, dCD-VQE folded proteins of up to nine amino acids using 17 qubits, achieving high success probabilities with low-depth circuits. Other quantum-enhanced sampling techniques are also emerging; for example, algorithms for antimicrobial peptides that include environmental effects such as membranes have been demonstrated without requiring additional qubits by adjusting the energy function (Conde-Torres et al., 2024) [30].

Studies comparing QuPepFold’s CVaR-VQE approach with brief molecular dynamics simulations have shown that the quantum method can sample lower-energy states more efficiently and avoid traps that MD can fall into [12,31]. By aggregating only the lowest-energy measurement outcomes, CVaR improves the chances of identifying the true ground state amid noisy measurements, though at the cost of extensive circuit evaluations.

Variational algorithms can suffer from barren plateaus and local minima, but focusing on the tail of the energy distribution via CVaR can sharpen convergence. QuPepFold has demonstrated stable convergence for seven-residue peptides [12,31], whereas shallow QAOA often required circuit depths beyond current hardware capabilities to surpass random guessing. The dCD-VQE algorithm further improves convergence through problem-inspired ansätze, achieving success probabilities above 90% for nine-mer proteins with lower circuit depths.

To validate the QuPepFold framework, we deployed the hybrid quantum-classical pipeline across a dataset of 1224 unique peptide sequences ranging from 6 to 10 residues in length. The system successfully generated and evaluated a massive ensemble of **1,761,280 distinct conformers**. This high-throughput generation confirms the stability of the backend integration, specifically the ability of the CVaR-optimized VQE to consistently converge on valid lattice structures without engaging in infinite loops or invalid topological clashes.For six-residue peptides, 244 sequences were tested. The method was highly successful: 99.6 percent of sequences reached a negative-energy fold, indicating that the allotted computational budget sufficed to find stable structures. The mean best energy was –2.97 kcal/mol, and the algorithm-maintained diversity by exploring roughly two distinct geometries per sequence.

For seven-residue sequences, 258 cases were examined and yielded the most stable results. The mean best energy dropped to –5.57 kcal/mol, and 97.3 percent of sequences found a negative-energy minimum. At this length, the algorithm effectively located the lowest-energy basins—about one distinct geometry per sequence—in approximately 7.5 seconds per sequence.

In the eight-residue set (172 sequences), the search space grew substantially. The mean best energy became positive (57.21 kcal/mol) under the limited sampling budget, but 13.4 percent of sequences still reached negative energies. This subset shows that QuPepFold can find stabilised folds even in larger spaces, although most runs end in positive-energy states. The search typically collapsed to a single dominant geometry, explaining about three-quarters of the energy variance.

For nine-residue peptides, 150 sequences were analysed and the mean energy was 63.80 kcal/mol. Although most low-budget runs produced positive energies, the method is expected to identify negative-energy folds in roughly 4–5 percent of cases. The correlation between geometry and energy became stronger (R² ≈ 0.81), implying that obtaining the correct geometry is the main obstacle to achieving negative energies.

For ten-residue sequences (135 cases), the mean energy was 70.45 kcal/mol and the success rate for finding negative energies under the current budget was about 1.5 percent. Even though positive-energy states dominate, the rare negative-energy instances demonstrate that the quantum-hybrid pipeline can theoretically locate the global minimum even when the landscape is filled with high-energy decoys.

A critical component of our analysis was distinguishing between energies derived from steric constraints (geometry) versus those derived from residue-specific interactions (modified Miyazawa–Jernigan potential). We identified 9,420 unique geometric topologies across the dataset. Statistical analysis yielded an R2 value of 0.676 when correlating geometry to total energy. This indicates that while 67.6% of the energy variance is governed by the structural topology of the backbone, a significant 32.4% is driven by the specific amino acid sequence. This finding is crucial, as it demonstrates that QuPepFold is sensitive to specific mutations and is not merely solving a geometric packing problem.

## 5 Conclusion

The quantum resources and circuit complexity required by QuPepFold increase rapidly with peptide length, limiting current applications to chains of about ten residues when using physics‑based potentials. Each additional residue expands the conformational space exponentially, and VQE sampling can entail millions of circuit shots. Alternative approaches provide different trade‑offs: for example, QFold’s quantum walk algorithm offers polynomial‑time exploration in theory but is currently hampered by hardware error rates and the need for repeated experiments, while the digitized‑counterdiabatic (dCD) method shortens circuits but still requires iterative parameter tuning.

Despite these challenges, QuPepFold distinguishes itself through its usability and flexibility. Delivered as a pip‑installable package built on Qiskit, it offers a command‑line interface that lets researchers run quantum folding simulations without needing to program quantum circuits themselves. Users can adjust ansatz forms, measurement shot counts and output visualizations. In contrast, many competing methods remain prototype codes or numerical demonstrations without ready‑to‑use implementations.

Because QuPepFold is modular, it can evolve alongside quantum hardware. As devices improve, the framework could be extended to handle longer peptides or more sophisticated ansätze, incorporate realistic force fields or solvation effects, or simulate peptide–ligand interactions by modifying the Hamiltonian. This adaptability contrasts with the constraints of quantum annealers, which are restricted to quadratic unconstrained binary optimizations tied to specific hardware. Off‑lattice approaches such as QFold could ultimately be applied to larger proteins but would require close integration with classical neural networks for energy evaluation.

Quantum folding tools are particularly relevant for problems that are difficult for classical computation, such as modelling intrinsically disordered regions where a single stable structure does not exist. QuPepFold’s CVaR‑VQE strategy can sample multiple low‑energy states and avoid getting trapped in kinetic barriers, hinting at the possibility of mapping entire conformational ensembles rather than just one native state. However, its focus on ground‑state optimisation may overlook the full diversity of biologically relevant conformations in IDRs, which often demand ensemble‑level sampling.

For small peptides relevant to drug discovery or cell signalling, quantum folding simulations hold promise for near‑term applications. Combining QuPepFold‑generated structures with classical refinement or AI‑based predictions could improve structural screening. Research on antimicrobial peptides interacting with membranes has shown that quantum algorithms can predict transitions from disordered to helical forms that are crucial for biological function. Although QuPepFold currently targets peptides in solution, it lays the groundwork for future extensions that incorporate environmental factors.

The successful processing of over 1.7 million conformers highlights the robustness of the Python-based modular architecture. This confirms that hybrid architectures can bridge the gap between current Noisy Intermediate-Scale Quantum (NISQ) limitations and the need for biological scale.

QuPepFold’s strengths lie in its accuracy on small peptides, its ability to produce three‑dimensional models of peptide backbones and its effective use of NISQ devices through a CVaR‑optimised VQE. Its limitations—including very short sequence lengths (< 6 residues) hardware noise and the need for extensive sampling—are shared across the field [12,31]. As quantum hardware and algorithms mature, the package’s hybrid strategy and extensibility position it to remain at the leading edge of quantum‑assisted structural biology.
